# Molecular characterization of nodule worm in a community of Bornean primates

**DOI:** 10.1002/ece3.5022

**Published:** 2019-03-05

**Authors:** Liesbeth Frias, Danica J. Stark, Milena Salgado Lynn, Senthilvel Nathan, Benoit Goossens, Munehiro Okamoto, Andrew J. J. MacIntosh

**Affiliations:** ^1^ Primate Research Institute Kyoto University Inuyama Japan; ^2^ Cardiff School of Biosciences Cardiff University Cardiff UK; ^3^ Danau Girang Field Centre Lower Kinabatangan Wildlife Sanctuary Sabah Malaysia; ^4^ Wildlife Health, Genetic and Forensic Laboratory Kota Kinabalu Malaysia; ^5^ Sustainable Places Research Institute Cardiff University Cardiff UK; ^6^ Sabah Wildlife Department Kota Kinabalu Malaysia; ^7^ Institute for Tropical Biology and Conservation Universiti Malaysia Sabah Kota Kinabalu Malaysia

**Keywords:** genetic diversity, host–parasite interactions, *Oesophagostomum*, soil‐transmitted helminths, Strongylida

## Abstract

Strongyles are commonly reported parasites in studies of primate parasite biodiversity. Among them, nodule worm species are often overlooked as a serious concern despite having been observed to cause serious disease in nonhuman primates and humans. In this study, we investigated whether strongyles found in Bornean primates are the nodule worm *Oesophagostomum* spp., and to what extent these parasites are shared among members of the community. To test this, we propose two hypotheses that use the parasite genetic structure to infer transmission processes within the community. In the first scenario, the absence of parasite genetic substructuring would reflect high levels of parasite transmission among primate hosts, as primates’ home ranges overlap in the study area. In the second scenario, the presence of parasite substructuring would suggest cryptic diversity within the parasite genus and the existence of phylogenetic barriers to cross‐species transmission. By using molecular markers, we identify strongyles infecting this primate community as *O. aculeatum*, the only species of nodule worm currently known to infect Asian nonhuman primates. Furthermore, the little to no genetic substructuring supports a scenario with no phylogenetic barriers to transmission and where host movements across the landscape would enable gene flow between host populations. This work shows that the parasite's high adaptability could act as a buffer against local parasite extinctions. Surveys targeting human populations living in close proximity to nonhuman primates could help clarify whether this species of nodule worm presents the zoonotic potential found in the other two species infecting African nonhuman primates.

## 1 INTRODUCTION

Parasites infecting closely related host species, or host species with overlapping home ranges, are often assumed to be generalist parasites (Davies & Pedersen, 2008). Given their ability to infect multiple host species, they are also considered to be important threats to biodiversity conservation and public health (Cleaveland, Laurenson, & Taylor, 2001; Jones et al., 2008). This perspective, however, has been challenged in recent years, as many alleged generalist parasites have turned out to be specialists in an ecological sense despite using a phylogenetically widespread set of resources, that is, specialization not for host species but for specific resources shared among them (Agosta, Janz, & Brooks, 2010; Nyman, 2009). This suggests that host specificity is more of a dynamic trait moving along a continuum than a black and white delineation (Janz, Nyblom, & Nylin, 2001; Nosil, 2002).

Gastrointestinal nematodes are an example of such a group, being geographically widespread and having the potential to infect multiple host species. Those nematode species with direct life cycles pass infective stages into the external environment, where they can remain for extended periods of time and transmit through shared habitat use, without the need for direct contact between hosts. Strongylid nematodes, for example, are among the most commonly reported gastrointestinal parasites found in wild primates. Despite being common, strongylids such as *Oesophagostomum* spp. (Nematoda, Strongylida) are often overlooked as a serious concern despite having been shown to cause serious disease in nonhuman primates and humans (Krief et al., 2010; Polderman & Blotkamp, 1995; Terio et al., 2011, 2018).

Three species of nodule worm are known to infect both nonhuman primates and humans (Table 1; Blotkamp et al., 1993; Chabaud & Larivière, 1958; Polderman & Blotkamp, 1995). The first, *O. bifurcum*, was reported as an endemic parasite of human health concern in Togo and Ghana (Krepel, 1994; Polderman & Blotkamp, 1995; Polderman, Krepel, Baeta, Blotkamp, & Gigase, 1991), where its unusually high prevalence in the human population (Krepel, Baeta, & Polderman, 1992; Storey et al., 2000) and subsequent detection in sympatric nonhuman primates (Gasser, Gruijter, & Polderman, 2006; Krief et al., 2010; van Lieshout et al., 2005; Stewart & Gasbarre, 1989) led epidemiologists to question whether nonhuman primates acted as wild reservoirs for human infection. Molecular characterization of the parasite later showed that humans and nonhuman primates harbored distinct parasite strains, with interspecific transmission being rare or absent (de Gruijter, Ziem, Verweij, Polderman, & Gasser, 2004; van Lieshout et al., 2005).

**Table 1:** *Oesophagostomum* species infecting primates for which molecular data have been reported

*Oesophagostomum* spp.	Locality	Primate family		References
		Hominidae	Cercopithecidae	
*O. aculeatum*	Malaysian Borneo	Bornean orangutan (*Pongo pygmaeus*)	Long‐tailed macaque (*Macaca fascicularis*), Proboscis monkey (*Nasalis larvatus*), Silvered langur (*Trachypithecus cristatus*),	This study
	Japan		Japanese macaque (*Macaca fuscata*)	Ota et al. (2015)
*O. bifurcum*	Uganda	Chimpanzee (*Pan troglodytes*)	Olive baboon (*Papio anubis*), Gray‐cheeked mangabey (*Lophocebus albigena*), L'hoest monkey (*Cercopithecus lhoesti*), Red colobus (*Procolobus rufomitratus*)	Ghai, Chapman et al. (2014), Cibot et al. (2015) and Ota et al. (2015)
	Tanzania		Yellow baboon (*Papio cynocephalus*)	Ota et al. (2015)
	South Africa		Chacma baboon (*Papio ursinus*)	Ota et al. (2015)
	Ghana		Mona monkey (*Cercopithecus mona*)	Gasser et al. (1999) and van Lieshout et al. (2005)
			Olive baboon (*Papio anubis*)	van Lieshout et al. (2005)
	Togo	Human (*Homo sapiens*)		Romstad et al. (1997)
*O. stephanostomum*	Uganda	Chimpanzee (*P. troglodytes*), Human (*H. sapiens*)	Black and white colobus (*Colobus guereza*)	Ghai, Chapman et al. (2014), Cibot et al. (2015) and Ota et al. (2015)
	Gabon	Chimpanzee (*P. troglodytes*), Lowland gorilla (*Gorilla gorilla gorilla*)		Makouloutou et al. (2014)
	Tanzania	Chimpanzee (*P. troglodytes*)		Gasser et al. (1999)

The other two nodule worm species that infect primates are *O. stephanostomum* and *O. aculeatum*. The former has been reported to infect chimpanzees (*Pan troglodytes schweinfurthii*, Huffman et al., 1996; Huffman, Gotoh, Turner, Hamai, & Yoshida, 1997, Krief et al., 2010, Kooriyama et al., 2013) and gorillas (*Gorilla gorilla gorilla, *Makouloutou et al., 2014) in the wild, but also humans in areas where humans, chimpanzees and other nonhuman primates live sympatrically (Cibot et al., 2015; Ota et al., 2015). However, *O. stephanostomum* has not been detected in humans living in close proximity to gorillas in Gabon (Makouloutou et al., 2014) or bonobos (*Pan paniscus*) in the Democratic Republic of the Congo (Narat et al., 2015), leading some to suggest that strains with differential capacities to infect humans may exist (Ota et al., 2015). *O. aculeatum*, on the other hand, is the least studied of these nodule worms, with most records of infection coming from macaques in Asia (Japanese macaques [*M. fuscata*]: Hashimoto & Honjo, 1966, Arizono, Yamada, Tegoshi, & Onishi, 2012, MacIntosh, 2014). Despite the extensive human–nonhuman primate interface throughout Asia (Fuentes, 2006), there have been but a few reported human cases in the region, for example, in Indonesia (Siang & Joe, 1953), Brunei (Ross, Gibson, & Harris, 1989), and Malaysia (Karim & Yang, 1992), with little published information regarding the causative agent.

As is the case with other soil‐transmitted helminths of public health concern, the first challenge for diagnosis is species identification, as strongyle eggs have almost no diagnostically useful morphological features. Similarities in morphology and morphometric measurements of certain eggs make species‐specific diagnosis particularly difficult. Primate species can be infected with several nematodes of the suborder Strongylida, such as hookworms (*Ancylostoma*, *Necator*), *Trichostrongylus*, *Ternidens,* and *Oesophagostomum*, whose eggs cannot be distinguished with certainty by microscopy alone (Polderman & Blotkamp, 1995). For example, there is considerable overlap in egg morphology and size for *Necator* and *Oesophagostomum*, but the former is significantly more pathogenic than the latter. Although morphology of infective‐stage L_3_ larvae is suitable for genus‐level identification in strongylid nematodes (Blotkamp et al., 1993), coprocultures are not routinely used for diagnosis in field studies, where parasite stages shed in host feces are typically the targets of screening efforts.

To overcome the limitations of morphological identification, there has been an increase in the use of molecular markers for clinical diagnostics and epidemiological studies, which have resulted in the identification of new lineages/strains within parasite species, showing new levels of diversity and transmission dynamics and suggesting that not all hosts are infected equally. In Uganda, two species of *Oesophagostomum* and a third uncharacterized and potentially zoonotic lineage have been reported to circulate in nonhuman primate communities and adjacent human populations (Cibot et al., 2015; Ghai, Chapman, Omeja, Davies, & Goldberg, 2014). A similar scenario has been documented for *Trichuris* spp., a whipworm infecting both humans and nonhuman primates in the same community (Ghai, Simons et al., 2014). For Bornean primates, we recently reported hidden patterns of diversity in *Strongyloides* spp., a neglected soil‐transmitted helminth infecting all members of a primate community (Frias et al., 2018). Thus, molecular diagnostics have the potential to vastly improve our understanding of parasite biodiversity in primate communities.

Here, toward a more complete picture of *Oesophagostomum* species infecting nonhuman primates in Asia, we aimed to examine the genetic diversity of the genus within a community of primates in the Lower Kinabatangan Wildlife Sanctuary, Malaysian Borneo. Although strongylid nematodes have been identified in several members of this community through microscopic detection of eggs in feces (Salgado Lynn, 2010, Klaus et al., 2017; L. Frias, H. Hasegawa, D. J. Stark, M. Salgado‐Lynn, S. Nathan, T. H. Chua, K. Keuk, B. Goossens, M. Okamoto, & A. J. J. MacIntosh, unpublished data), their specific identities remain unknown. Therefore, we set out to investigate whether strongylid infection in Bornean primates is in fact caused by *O. aculeatum* and to what extent the parasite is being shared across the primate host community. We tested two competing but not necessarily mutually exclusive hypotheses with our data set. First, overlapping ranges among primate hosts are suspected to be high in the area, which can facilitate panmixia (i.e., random mating or homogenization among parasite populations) in environmentally transmitted parasites such as nodule worm. We therefore predicted that there would be little or no genetic substructuring in the parasite. However, we also hypothesized that, if substructuring is evident, phylogenetic barriers may be the most likely explanation. In the latter case, we predicted that substructuring in the parasite population would occur between rather than within primate lineages.

## 2 MATERIALS AND METHODS

### 2.1 Study site and sample collection

The Lower Kinabatangan Wildlife Sanctuary is located in the eastern region of Sabah (Malaysian Borneo) and covers an expanse of 15,000 ha of natural forest types, including dry lowland, semi‐inundated, semi‐swamp/grassy and swamp forests, and lands that have been cleared for oil‐palm development or repeatedly logged in the past (Ancrenaz, Calaque, & Lackman‐Ancrenaz, 2004). Ten primate species are known to inhabit the area, including long‐tailed and southern pig‐tailed macaques (*Macaca fascicularis*, *M. nemestrina*), silvered, maroon and Hose's langurs (*Trachypithecus cristatus*, *Presbytis rubicunda* and *P. hosei*), proboscis monkeys (*Nasalis larvatus*), Bornean orangutans (*Pongo pygmaeus*), Bornean gibbons (*Hylobates muelleri*), Western tarsiers (*Cephalopacus bancanus*), and Philippine slow lorises (*Nycticebus menagensis*). In October 2015, we conducted noninvasive fecal sampling of the most abundant species within three groups of primates: colobines (proboscis monkeys and silvered langurs) and macaques (long‐tailed macaques), mostly occupying riparian zones, and orangutans inhabiting areas within the forest. Samples from macaques and colobines were collected from the ground around sleeping sites along the riverbanks between 6:00 a.m. and 8:00 a.m. As direct observation of defecation was not possible with these unhabituated groups, which often have overlapping ranges, we evaluated feces according to their softness and the presence of insects, such as dung beetles or carrion flies to maximize the collection of fresh feces. Orangutans, on the other hand, were tracked in the forest, where fecal samples were collected immediately after defecation.

Although short fragments of strongyle parasite DNA can be detected in host feces using PCR (de Gruijter, Gasser, Polderman, Asigri, & Dijkshoorn, 2005; Gasser et al., 2006; Verweij, Polderman, Wimmenhove, & Gasser, 2000), such short sequences may obscure variability within a given gene and thus longer sequences are preferred. From the collected fecal samples, we set up coprocultures (*N* = 24, silvered langurs (7), proboscis monkeys (7), long‐tailed macaques (4), and orangutans (6)) using a modified Harada‐Mori filter‐paper technique following Hasegawa (2009) to develop L_3 _larvae and extract DNA directly from them instead. Developed larvae migrating into the water were fixed in 70% ethanol and later morphologically identified to genus level using standard keys (Anderson, Chabaud, & Willmott, 2009).

### 2.2 DNA extraction and phylogenetic analyses

As many samples were not collected after observing defecation, and most primate species have overlapping ranges in the area, we conducted host species identification for all samples used in coprocultures by extracting whole DNA from each collected fecal sample and amplifying a small fragment of the *cytochrome b* (*cytb*) gene, following the protocol described in Frias et al. (2018). Then, 64 strongylid larvae were individually selected at random for DNA extraction (Table 2) using a QIAamp DNA micro kit (Qiagen, Japan). PCRs were conducted targeting a fragment of the mitochondrial *cytochrome c oxidase subunit 1* (*cox1*) gene using the primers StrCoxAfrF (5′‐GTGGTTTTGGTAATTGAATGGTT‐3′) and MH28R (5′‐CTAACTACATAATAAGTATCATG‐3′) (Hasegawa et al., 2010; Hu, Chilton, & Gasser, 2003). Each PCR was carried out in 15 μl reaction mixtures containing 1.5 μl template, 200 μM of each dNTP, 5 μM of each primer, 0.5 U of Ex‐Taq polymerase (Takara), and the manufacturer supplied reaction buffer. Thermal reaction was performed under an initial denaturation at 94°C for 2 min, followed by 30 cycles of 98°C for 15 s, 45°C for 30 s, and 68°C for 30 s, and a final extension at 68°C for 5 min. Following PCR amplification, nonspecific products were removed from the amplicons using the Agencourt AMPure system (Agencourt Bioscience Corp., Beverly, MA) and aliquots were sequenced in an ABI‐PRISM 3,130 Genetic Analyzer (Applied Biosystems, CA, USA). Nucleotide sequences were aligned and minimally adjusted in MEGA 6.06 (Tamura, Stecher, Peterson, Filipski, & Kumar, 2013). To provide phylogenetic context to the analysis, we included published sequences of *cox1* from *Oesophagostomum* spp. infecting several primate species. Phylogenetic trees were also reconstructed in MEGA, using maximum likelihood (ML) and neighbor‐joining (NJ) algorithms with bootstrap values calculated using 1,000 replicates. Sequences obtained in this study were deposited in the DNA Database of Japan (DDBJ) under accession numbers LC428761‐LC428824.

**Table 2:** *Oesophagostomum* spp. larvae analyzed in relation to their distribution in four species of Bornean primates in the Lower Kinabatangan Wildlife Sanctuary

Primate species	Culture ID (*N* = 24)	Strongyle larvae^a^(*N* = 64)	Molecular identification
Long‐tailed macaques (*M. fascicularis*)	Mf1	11	*O. aculeatum *(11)
	Mf2	10	*O. aculeatum *(10)
	Mf3	1	*O. aculeatum *(1)
	Mf4	1	*O. aculeatum *(1)
Silvered langurs (*T. cristatus*)	Tc1	0	
	Tc2	0	
	Tc3	2	*O. aculeatum *(2)
	Tc4	0	
	Tc5	0	
	Tc6	0	
	Tc7	0	
Proboscis monkeys (*N. larvatus*)	Nl1	0	
	Nl2	0	
	Nl3	1	*O. aculeatum *(1)
	Nl4	0	
	Nl5	0	
	Nl6	0	
	Nl7	0	
Bornean orangutans (*P. pygmaeus*)	Pp1	17	*O. aculeatum *(17)
	Pp2	5	*O. aculeatum *(5)
	Pp3	13	*O. aculeatum *(12)/*T. deminutus *(1)
	Pp4	3	*O. aculeatum *(3)
	Pp5	0	
	Pp6	0	

a Not identified to genus level.

### 2.3 Ethics statement

Authorization to conduct research in Sabah, collect samples, and export them to Japan was provided by the Sabah Biodiversity Centre (SaBC) and the Sabah Wildlife Department. Our field protocols also adhered to the guidelines set by these agencies.

## 3 RESULTS

The overall prevalence of strongyles in the studied community was low to moderate (38%), and although most fecal samples used for coprocultures turned out to be strongyle‐negative by microscopy, we were still able to successfully develop L_3_ larvae in 10 of them (Table 2). Strongylid larvae were identified from orangutans (*N* = 37), long‐tailed macaques (*N* = 24), silvered langurs (*N* = 2), and proboscis monkeys (*N* = 1), for a total of 64 isolates. Strongyle prevalence and EPG (egg per grams of feces) counts, as determined through microscopy (L. Frias, H. Hasegawa, D. J. Stark, M. Salgado‐Lynn, S. Nathan, T. H. Chua, K. Keuk, B. Goossens, M. Okamoto, & A. J. J. MacIntosh, unpublished data), varied among primate species with the highest prevalence observed in orangutans (42.8%) and the highest mean EPG‐abundance observed in colobines (413 ± 1,028 for silvered langurs, and 835 ± 1,423 for proboscis monkeys).

**Figure 1:**
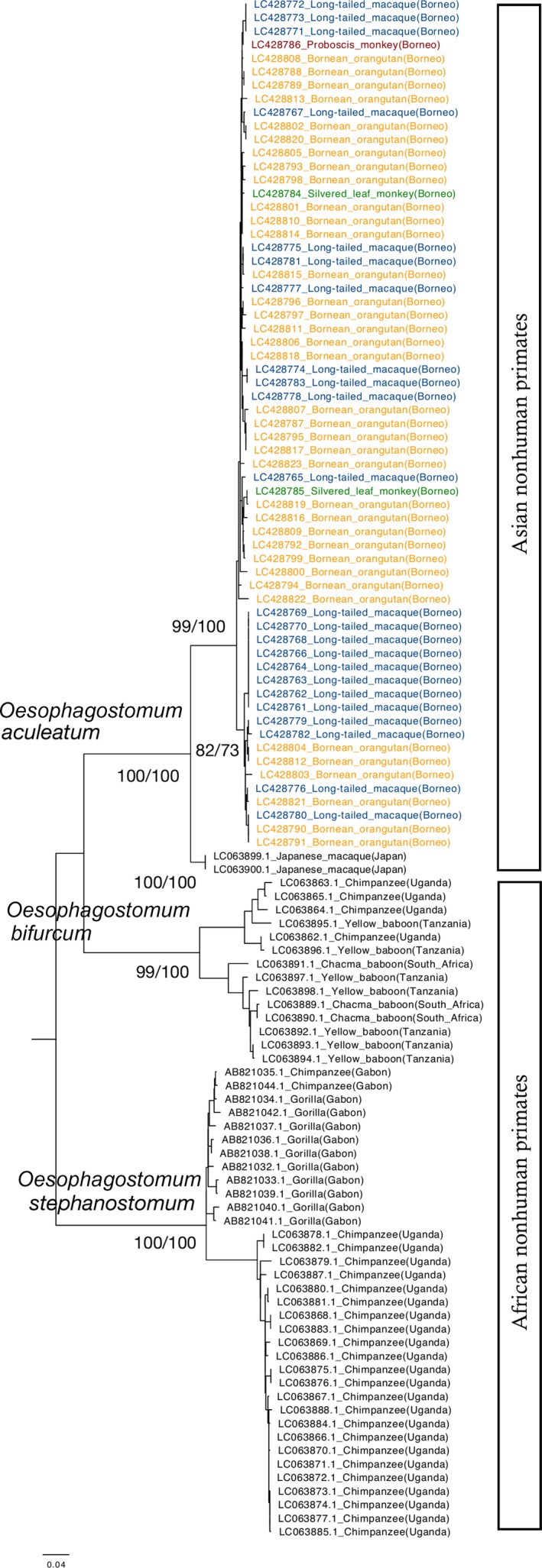
Phylogenetic relationships among *Oesophagostomum* species infecting primates inferred from *cox1* gene sequences (only ML tree shown). Branch support is represented by ML/NJ bootstrap values, respectively

Partial *cox1* was sequenced from all larvae, resulting in 63 sequences of 823 bp closely matching those published for *O. aculeatum*. As one isolate resulted in an unidentified sequence, we further sequenced the second inter transcribed spacer of the rDNA (ITS2), using the primers described in Gasser, Woods, Huffman, Blotkamp, and Polderman (1999), obtaining a sequence of 216 bp identified as *Ternidens deminutus*. Phylogenetic analysis of *O. aculeatum* isolates consisted of an alignment of 114 sequences trimmed to 766 bp to ensure comparison among homologous regions of the gene. Mean intra‐species variation for *O. aculeatum*, *O. stephanostomum,* and *O. bifurcum* amounted to 1.8%, 1.9% and 8.9% respectively, while overall mean interspecies variation was 9.9%. NJ and ML trees resulted in similar topologies; therefore, only the ML tree is presented (Figure 1). The phylogenetic tree shows a first split distinguishing *O. stephanostomum* from *O. aculeatum* and *O. bifurcum* (Figure 1). The *O. stephanostomum* clade clustered all great apes from Gabon into one group, and chimpanzees from Uganda in the other, showing a clear geographic structure. The *O. bifurcum* clade grouped baboons, regardless of their origins, in one cluster, and both baboons and chimpanzees from West Africa in the other. Finally, the *O. aculeatum* clade split into those parasites infecting Japanese macaques and those infecting Bornean primates, represented by two clusters. Samples from orangutans and long‐tailed macaques are spread throughout the Bornean group, while samples from colobines are only present in one of them. It should be noted here that the colobine data comprised only three individual larvae from three fecal samples, so these results should be interpreted with caution until further data are available.

## 4 DISCUSSION

Studies on sympatric host species offer a good opportunity to understand the role of species heterogeneity in a transmission process involving parasites acquired through shared habitat use, as certain species will be more susceptible than others to infection or more likely to contribute to parasite transmission (Morgan, Milner‐Gulland, Torgerson, & Medley, 2004). In contrast to the advances in understanding transmission dynamics of *Oesophagostomum* species infecting human and nonhuman primates in Africa, we still know little about *O. aculeatum*, the only species presumed to infect nonhuman primates and humans in Asia (Stewart & Gasbarre, 1989). Our study thus investigated its patterns of diversity in a multi‐host, multi‐parasite system in Malaysian Borneo, in an area where several primate species are known to live sympatrically and to be infected by strongylid nematodes across their range. Through coprocultures and genetic characterization of L_3_ larvae, we showed that *O. aculeatum* is widely distributed in the study area and that it infects all studied members of the community with little clear genetic differentiation among them.

As parasitological surveys of wild primates tend to provide coarse information at best, we generally lack information regarding not only which parasites are infecting primates but also how they may be impacting their host populations. In humans, the clinical manifestation of oesophagostomiasis is generally associated with the presence of nodules (encysted L_4_ larvae) in the intestinal mucosa (Polderman & Blotkamp, 1995). *Oesophagostomum* spp. infection in communities of African primates at Mahale, Kibale, and Taï has revealed that not only is the parasite detectable in feces (Ghai, Chapman et al., 2014; Kooriyama et al., 2013; Kouassi et al., 2015), but it may have consequences for individual health. Clinical signs associated with the onset of disease have been observed in both chimpanzees and baboons (*Papio anubis*), and chimpanzees have been observed swallowing whole leaves to purge intestinal worms (Huffman & Caton, 2001; Huffman et al., 1996; Terio et al., 2011, 2018). Given that *Oesophagostomum* spp. and other strongylid nematodes are known to impact health and fitness in a broad range of nonprimate hosts as well (Roepstorff, Bjørn, Nansen, Barnes, & Christensen, 1996; Waghorn, Bouchet, Bekelaar, & Leathwick, 2019), efforts to better understand its distribution and diversity are warranted.

In general, although* Oesophagostomum* spp. seem to have the potential to infect all or at least many members of primate communities, the parasite's ability to infect each host species and its fitness in each one of them remains to be explored. For example, *Oesophagostomum* spp. infection in colobines suggests different host–parasite dynamics than that observed in chimpanzees. A recent study looking at *Oesophagostomum*‐associated pathology in sympatric primates at Gombe reported that, in contrast to chimpanzees and baboons that showed high numbers of nodules, none were found in red colobus (*Procolobus rufomitratus*) also inhabiting the area (Terio et al., 2018). The effect of diet on the establishment and persistence of the parasite could be a venue for future exploration in this regard. It has been suggested that diets with high levels of insoluble digestible fiber, such as wheat bran, whole grains, and cereals, favor *O. dentatum* survival, growth, and reproduction in farm pigs, leading to a significant increase in the parasite's infection intensity (Petkevičius, Nansen, Knudsen, & Skjøth, 1999). Colobines’ low‐digestible diet, consisting mostly of leaves, seeds, and unripe fruit (Chivers, 1994) may have a regulatory function in the parasite's ecology.

*Oesophagostomum aculeatum* was not the only strongylid nematode infecting Bornean primates observed in this study. We were also able to genetically identify a single isolate of *Ternidens deminutus* (Nematoda, Strongylida) from a Bornean orangutan. *T. deminutus* is an understudied nematode infecting primates and has been regarded as a zoonotic parasite able to affect human health (Hemsrichart, 2005). Given the shared morphology of their eggs, if *T. deminutus* co‐occurs with other strongylid nematodes, such as hookworms or other species, its presence can be easily overlooked in coprological analysis for parasite evaluation in primates, much like *Oesophagostomum* spp. *Ternidens deminutus* has been genetically characterized in olive baboons and mona monkeys (*Cercopithecus mona*) in Ghana (Schindler, Gruijter, Polderman, & Gasser, 2005), and long‐tailed macaques in China (direct submission to GenBank). Unfortunately, at present, we still know very little about its biology, transmission, or the extent of its effects on primate hosts.

In conclusion, our results show a widespread distribution of *O. aculeatum* in this primate community, in agreement with previous observations of a different soil‐transmitted helminth (*Strongyloides* spp.) infecting the same community (Frias et al., 2018), further suggesting that extensive habitat overlap in primates may be contributing to the little parasite genetic substructuring observed. Continuous parasite gene flow between host species suggests a high transmission potential enabled by host movement (Blouin, Yowell, Courtney, & Dame, 1995). From a metapopulation perspective, the dispersal abilities of parasite species through host movement play a key role in parasite local adaptation and the resilience of host–parasite associations to habitat disturbance, providing a buffer against local parasite extinctions (Gandon & Michalakis, 2002; Legrand et al., 2017; Thompson & Gonzalez, 2017). Finally, although our sampling and analyses did not include the human population, we do not discard the possibility of *O. aculeatum* having a zoonotic potential in the same fashion that *O. bifurcum* and *O. stephanostomum* do. Strongyle‐like eggs are often assigned to hookworm eggs in areas where the latter represent an infection of public health concern. Hookworm has been reported in surveys looking at helminthiases infecting children in rural areas and indigenous communities in West and East Malaysia (Anuar, Salleh, & Moktar, 2014; Huat et al., 2012; Mohd‐Shaharuddin, Lim, Hassan, Nathan, & Ngui, 2018; Rajoo et al., 2017), but they are often not assigned to parasite species (but see Ngui, Lim, Traub, Mahmud, & Mistam, 2012, Sahimin et al., 2017). Surveys targeting human populations living in close proximity to nonhuman primates would go a long way toward exploring that possibility.

## CONFLICT OF INTEREST

None Declared.

## AUTHOR CONTRIBUTIONS

LF, MO, and AJJM designed the research. LF and AJJM collected the data. LF conducted the laboratory work and data analysis. LF wrote the manuscript with input from MO and AJJM. DJS, MSL, SKSSN, and BG provided logistical support and comments to the manuscript.

## Data Availability

All DNA sequences obtained in this study were deposited in DDBJ/GenBank, under Accession Numbers LC428761‐LC428824.
